# The Inactivation of Hippo Signaling Pathway Promotes the Development of Adenomyosis by Regulating EMT, Proliferation, and Apoptosis of Cells

**DOI:** 10.1007/s43032-023-01189-w

**Published:** 2023-03-20

**Authors:** Tingting Jin, Mengqi Li, Ting Li, Simiao Yan, Qingzhen Ran, Wanqun Chen

**Affiliations:** 1 Department of Biochemistry and Molecular Biology, School of Medicine, Jinan University, Guangzhou, 510632 Guangdong China; 2 Department of Gynecology, Guangdong Provincial Hospital of Chinese Medicine, Guangzhou, 510120 Guangdong China; 3 Engineering Technology Research Center of Drug Development for Small Nucleic Acids, Guangzhou, 510632 Guangdong China

**Keywords:** Adenomyosis (ADM), YAP (Yes-associated protein), Hippo signaling pathway, Epithelial-mesenchymal transition (EMT)

## Abstract

Adenomyosis is a benign gynecological disease. The pathogenesis of adenomyosis is still unclear. The Hippo signaling pathway is highly conserved in vivo and associated with endometriosis and various cancers. Our objective was to study the expression of Hippo signaling pathway–related proteins in the uterus of mice with and without adenomyosis. We also sought to determine the relationship between the Hippo signaling pathway and cell migration, invasion, proliferation, and apoptosis in adenomyosis. The inactivation of Hippo signaling pathway and abnormal expression of EMT-related proteins were observed in mice with adenomyosis. In vitro, the YAP inhibitor verteporfin can inhibit the proliferation and migration of Ishikawa cells and promote apoptosis, while inhibiting the EMT process. In addition, intraperitoneal injection of verteporfin inhibits EMT process and proliferation and promotes apoptosis of cells in the uterus of adenomyosis mice. It suggests that the Hippo signaling pathway participates in the EMT, proliferation, and apoptosis of cells in adenomyosis. In conclusion, these results suggest that Hippo signaling pathway may be involved in the development of adenomyosis by regulating EMT, proliferation, and apoptosis of cells, which provide a potential target for the treatment of adenomyosis.

## Introduction

Adenomyosis is a common benign chronic gynecological disease [1, 2], which is characterized by abnormal growth of glands and stroma invading into the myometrium, accompanied with hypertrophy and hyperplasia of the myometrium [3]. Common clinical symptoms include increased uterine diffusion, dysmenorrhea, pelvic pain, abnormal uterine bleeding (AUB), and infertility [4], which seriously affect the quality of life of the patients. Currently, the pathogenesis of adenomyosis is still unclear, but one of the generally accepted theories is the invagination theory, in which endometrial cells invade the myometrium by acquiring invasiveness [5, 6]. Previous studies have demonstrated that epithelial-mesenchymal transition (EMT) plays an important role in cell migration and invasion and is associated with the development of adenomyosis [7, 8].

EMT refers to the process in which epithelial cells undergo phenotypic transformation under specific conditions and acquire a mesenchymal phenotype with stronger migration ability [9]. The occurrence of EMT will promote the enhancement of cell invasion and migration ability, and the existence of EMT can be found in various malignant tumors [10]. The typical molecular feature of EMT is that epithelial cells lose the expression of the epithelial marker (E-cadherin) and acquire the expression of mesenchymal markers (such as N-cadherin and Vimentin) [9]. In addition, a variety of transcription factors can also promote the occurrence of EMT by inhibiting the expression of E-cadherin, such as twist basic helix-loop-helix transcription factor (Twist), snail family zinc finger 2 (Slug), and snail family zinc finger 1 (Snail) [11]. Several studies have reported the role of EMT in adenomyosis. Chen et al. [8] reported for the first time that estrogen-induced EMT is involved in the development of adenomyosis. In addition, HGF-induced EMT may also be involved in the invagination of glands into the myometrium in adenomyosis [12].

The Hippo signaling pathway was originally discovered to regulate the size of tissues and organs in Drosophila [13]. The core components of the pathway include mammalian Ste20-like 1/2 (Mst1/2) and large tumor suppressor 1/2 (Lats1/2) regulating transcriptional coactivators, yes-associated protein (YAP), and transcriptional coactivator with PDZ-binding domain (TAZ) [14]. As a key protein of Hippo signaling pathway, the abnormal regulation of YAP is related to the occurrence and development of various cancers [15, 16]. When YAP is phosphorylated, it is sequestered in the cytoplasm by binding to 14–3-3 protein and then degraded [17]; when YAP is not phosphorylated, it can enter the nucleus and combine with the transcription factor TEA domain family members (Teads) to promote gene transcription to regulate cell proliferation, migration, and invasion [18]. Although overexpression of YAP has been found in uterine endometrial-myometrial junctional zone (JZ) tissues of patients with adenomyosis [19], there is still a lack of corresponding research on its molecular mechanism in the pathogenesis of adenomyosis. Previous studies have shown that the occurrence of EMT is often accompanied with the up-regulation of YAP expression and the increase of YAP content in the nucleus [20]. Therefore, we speculate that YAP is involved in the pathological development of adenomyosis by inducing the occurrence of EMT.

Currently, we aimed to study the change of Hippo signaling pathway and its effect on proliferation, apoptosis, and EMT of cells in adenomyosis. On the basis of verifying that activating the Hippo signaling pathway can inhibit the proliferation, migration, and EMT of endometrial cells and promote apoptosis, we further demonstrated in vivo experiments that abnormal inactivation of the Hippo signaling pathway occurred in the uterus of mice with adenomyosis and demonstrated that the YAP inhibitor verteporfin can inhibit EMT and proliferation and promote apoptosis of cells in the uterus of mice with adenomyosis. Therefore, our study may provide a new idea for exploring the pathogenesis of adenomyosis.

## Materials and Methods

### Cell Culture and Drug Treatment

The Ishikawa cells (Human Asia endometrial adenocarcinoma cell line) were derived from a 39-year-old female with endometrial adenocarcinoma. They are adherent epithelioid cells expressing ER and PR. Prominent gene expression changes in Ishikawa cells mimic physiological processes in normal endometrial gland cells, and this cell line is often used as a model of reproductive disorders including adenomyosis [21]. The Ishikawa cells were cultivated in DMEM/F12 containing 10% fetal bovine serum (FBS). Verteporfin (MedChemExpress), a YAP inhibitor, was dissolved in dimethyl sulfoxide (DMSO) and stored at − 20 °C. In vitro, Ishikawa cells were treated with fresh medium or different concentrations of verteporfin.

### CCK-8 Assay

We used CCK-8 to detect cell proliferation. Briefly, 100 μl of cell suspension containing 5 × 10^3^ cells was seeded in 96-well plate and cultured for 24 h. Cells were treated with different concentrations of verteporfin for 24 h. Then, 10 μl of CCK-8 solution was added to each well and incubated at 37 °C for 4 h. The absorbance at 450 nm was measured with a microplate reader.

### Colony Formation Assay

Ishikawa cells were seeded in six-well plates at a density of 500 cells/well. Thereafter, the cells were cultured for 14 days to form colonies, during which time the medium was changed every 3 days. After that, cells were fixed with 4% paraformaldehyde for 30 min and stained with 0.1% crystal violet.

### Wound Healing Assay

Wound healing assay was used to detect cell migration. Ishikawa cells were seeded in six-well plates at a density of 5 × 10^5^ cells/well. After culturing the cells until the confluence reaches 80–90%, 200 μl pipette tips were used to draw a straight line. Excess cells were washed with phosphate buffered saline (PBS). The cells were cultured in serum-free medium. The scratch position was observed and photographed under the microscope at 0, 6, and 12 h, respectively. Images were analyzed with Image J.

### Transwell Migration Assay

Transwell migration assay was used to detect cell migration. Five hundred microliters of DMEM/F12 medium containing 10% FBS was added to a 24-well plate. After placing the chamber, 5 × 10^4^ cells suspended in serum-free medium were seeded in the chamber. After culturing for 24 h, the cells remaining in the upper chamber were gently removed with a cotton swab. After fixation with 4% paraformaldehyde, the cells that penetrated the membrane were stained with 0.1% crystal violet solution. Stained cells were counted under a light microscope.

### 5-Ethynyl-2′-Deoxyuridine (EdU) Assay

Cell proliferation was probed by EdU detection kit (KeyGen Biotech, China). Verteporfin-treated Ishikawa cells were incubated with 10 μM EdU reagent for 3 h. Cells were then fixed with 4% paraformaldehyde and treated with 0.5% Triton-X-100 to permeabilize cells. Nucleic acids were stained with DAPI after 0.5 h staining with the Click-iT reaction mix. Finally, EdU positive cells were counted and photographed with a fluorescence microscope (Leica, Germany).

### Flow Cytometry Analysis

Apoptotic cells were detected by using the Annexin-V-APC/PI double staining kit (KeyGen Biotech, China). After detaching the verteporfin-treated cells from the six-well plate with 0.25% trypsin (without EDTA) (Gibco, USA), they were washed twice with PBS and centrifuged (2000 g, 5 min). Cells were then resuspended in binding buffer and stained with Annexin-V-APC and PI for 30 min in the dark. Finally, we detected stained cells by using flow cytometry and analyzed apoptosis with FlowJo X software.

### Animals and Experiment Protocol

All experiments were performed in accordance with the guidelines of the National Research Council’s *Guide for the Care and Use of Laboratory Animals* and approved by the Laboratory Animal Review Committee of Jinan University (Approval number: IACUC-20200905–01).

We established the mouse model of adenomyosis by using tamoxifen [22]. Six female (7-week-old) and three male (8-week-old) ICR mice were purchased from Beijing Huafukang Company. Under controlled conditions (20 °C, 12-h light–dark cycle), two female mice and one male mouse were housed in the same cage with ad libitum access to water and food. After the female mice became pregnant, each female mouse was housed in a separate cage, and the newborn mice were used for subsequent modeling experiments.

The neonatal female mice were randomly divided into two groups: tamoxifen (TAM) and control group. Mice in the TAM group were given oral tamoxifen to induce adenomyosis, and the control group was given only the vehicle in the same way. Mice in the TAM group were fed 1 mg/kg of tamoxifen suspended in a mixture of peanut oil/lecithin/condensed milk (2:0.2:3 by volume) at a dose of 5 µl/g body weight per day from day 2 to 5 of birth. At the same time, the control group was fed the same dose of solvent without tamoxifen. When female mice were grown to day 21, they were weaned and separated from dams.

Mice were housed under normal conditions for 60 days. Three mice were randomly selected from each of the control group and the TAM group and sacrificed. The mouse uterus were collected for paraffin embedding to make paraffin sections. Histological examination was performed by hematoxylin and eosin (H&E) staining to verify whether the modeling was successful.

After successful modeling, five mice were randomly selected from each of the control group and the TAM group as the control group and the adenomyosis group in the next phase of the study. The remaining mice were randomly divided into three groups: control group (*n* = 5), adenomyosis group (*n* = 5), and verteporfin group (*n* = 5). Mice in the verteporfin group were intraperitoneally injected with 50 mg/kg of verteporfin, and the control group and adenomyosis group were given the same dose of solvent. All mice were administered consecutively for 7 days and sacrificed by cervical dislocation on the first day after the last administration.

### Protein Extraction and Western Blot Analysis

Fresh tissues or cells were added to RIPA lysis buffer, sonicated, lysed on ice for 30 min, centrifuged at 12,000 g at 4 °C for 15 min to extract total protein, and quantified with BCA protein detection kit (Beyotime, China). Twenty micrograms of protein was loaded on an SDS-PAGE gel, which was then transferred to a PVDF membrane (Millipore, USA). After blocking in 5% nonfat milk (BD, USA) for 1 h at room temperature, the membrane was incubated with primary antibody overnight at 4 °C. Then, after washing off the primary antibody, the membrane was incubated with HRP-conjugated goat anti-rabbit or mouse secondary antibody for 1 h at room temperature. Protein expression was determined using a chemiluminescence system and analyzed with Imaje J software.

### Immunohistochemical Analysis

Paraffin sections were subjected to routine dewaxing and rehydration procedures. The sections were boiled with citric acid antigen retrieval solution (pH = 6.0) for 15 min for antigen retrieval. Endogenous peroxidase activity was removed by 3% H_2_O_2_. Ten percent goat serum was used to block nonspecific binding for 1 h at room temperature. Then, sections were incubated with primary antibodies overnight at 4 °C. After washing off the primary antibody, sections were then incubated with the secondary antibody for 2 h at room temperature. Immunoreactive proteins were visualized by using 3, 3-N-Diaminobenzidine tetrahydrochloride (Sangon Biotech, China). Sections were then counterstained with hematoxylin. Finally, sections were observed and photographed under an optical microscope (Nikon, TOKYO).

### TUNEL Assay

The apoptosis of mouse uterine tissue cells was detected by one-step TUNEL Apoptosis Detection Kit (Keygen Biotcch, China). After routine deparaffinization and hydration of sections, permeabilization of cells was increased with protease K (10 ×). Then, the sections were incubated with the TdT reaction mixture (Equilibration Buffer:biotin-11-Dutp:TdT Enzyme = 45:1:5) in a humidified chamber for 60 min at 37 °C. After PBS solution washing, the sections were incubated with Streptavidin-Fluoresce solution (Streptavidin-Fluoresce solution:Labeling Buffer = 1:9) for 30 min at 37 °C, away from light. The sections were then counterstained with DAPI for 10 min before sample analysis by fluorescence microscopy (Leica, Germany).

### Statistical Analysis

In this experiment, three independent replicates were performed. GraphPad Prism 8.0 software was used to process and draw experimental data. Data of repeated experiments are expressed as mean ± SD. Data statistics and analysis between two groups were compared using Student’s *t*-test, and three groups were analyzed and compared using one-way ANOVA test; *p* < 0.05 was regarded as significant difference.

## Results

### Inactivation of Hippo Signaling Pathway and Occurrence of EMT in Adenomyosis Mice

**Fig. 1:**
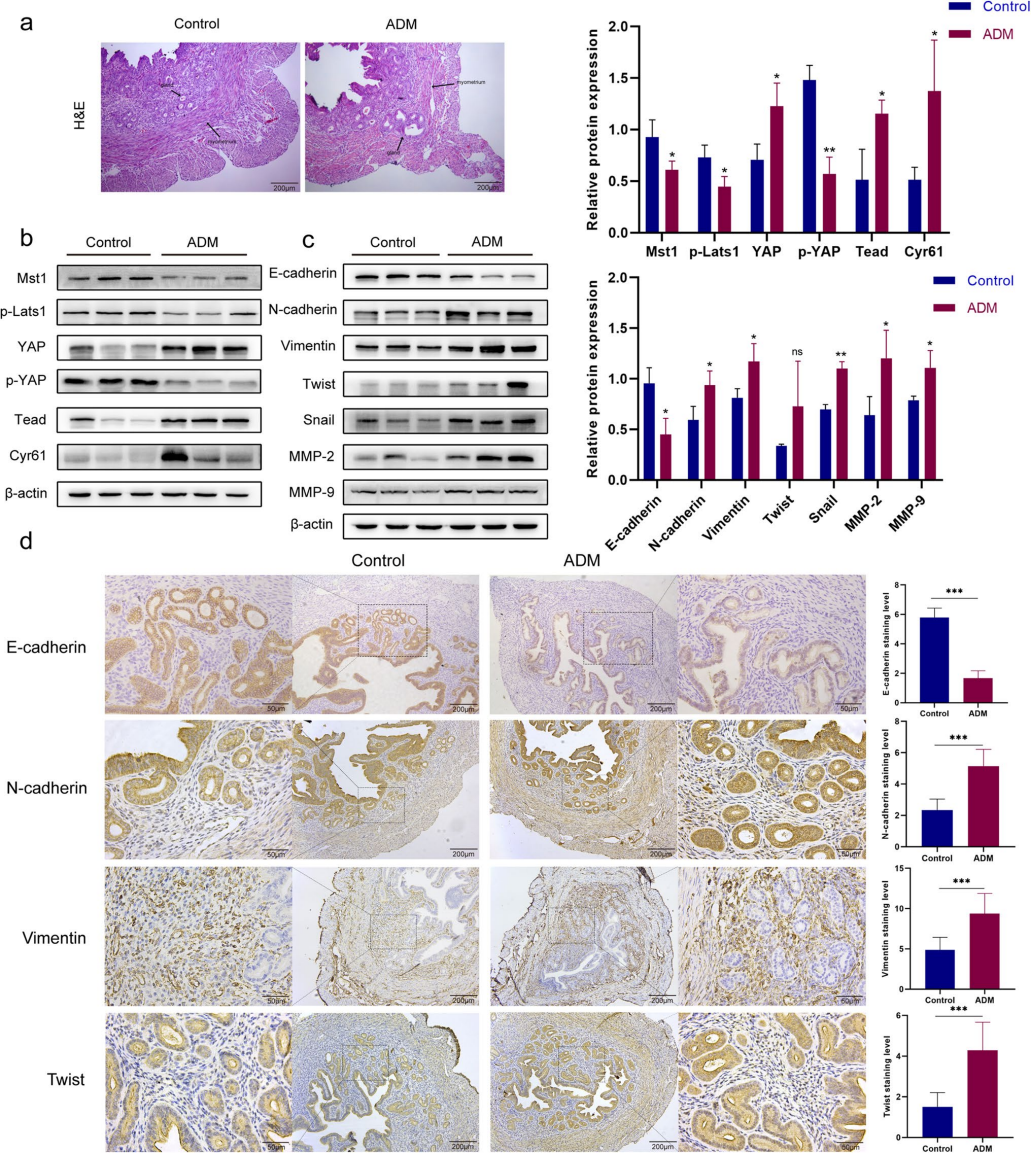
Inactivation of Hippo signaling pathway and occurrence of EMT in adenomyosis mice. **a** H&E staining of the successful establishment of adenomyosis mice. **b** Protein expression of Mst1, p-Lats1, YAP, p-YAP, Tead, and Cyr61 in uterine tissue was determined by western blot. **c** Protein expression of E-cadherin, N-cadherin, Vimentin, Twist, Snail, MMP-2, and MMP-9 in uterine tissue was determined by western blot. **d** The expression levels of E-cadherin, N-cadherin, Vimentin, and Twist in uterine tissue were detected by IHC assay. Data were presented as mean ± SD. *n* = 5. *P < 0.05; **P < 0.01; ns, no significance

In order to investigate the correlation of Hippo signaling pathway and EMT with adenomyosis, we established a mouse model of adenomyosis by instilling tamoxifen to newborn ICR mice. As shown in Fig. 1a, the results of H&E staining showed that the myometrium of the control group was arrayed in bundles and had been well-spaced from the endometrium, whereas the endometrium stroma from the TAM group was immersed into the muscular layer that was divided into multiple bundles by the stroma. In addition, in the sample from the TAM group, the smooth muscle structure of the muscular layer had been notably disordered, and the endometrium boundary appeared unclear. These findings illustrate that the establishment of adenomyosis mice is successful.

Then, we detected the expression levels of Hippo signaling pathway–related proteins by western blot. As shown in Fig. 1b, the expression level of YAP in adenomyosis mice was significantly higher and the p-YAP level was lower than that in control mice. In addition, the expression of other proteins of the Hippo signaling pathway, Mst1 and p-Lats1, was decreased, and the expression of Tead and YAP target gene Cyr61 was increased. These results suggest that abnormal inactivation of the Hippo signaling pathway occurs in mice with adenomyosis.

After demonstrating abnormal inactivation of Hippo signaling pathway in adenomyosis mice, we further investigated the expression of EMT-related proteins in adenomyosis by western blot and IHC analysis. As shown in Fig. 1c, the results of western blot showed that the expression of the epithelial marker E-cadherin decreased, and the expression of the mesenchymal markers N-cadherin and Vimentin increased in adenomyosis mice compared with control mice. Our study also detected the expression of multiple transcription factors that inhibit E-cadherin protein level. The expression of Twist and Snail was increased in adenomyosis mice compared with control mice. In addition, the expression of proteins matrix metallopeptidase 2 (MMP-2) and matrix metallopeptidase 9 (MMP-9) related to invasion and migration was elevated. Consistently, the expression levels of E-cadherin, N-cadherin, Vimentin, and Twist detected by IHC further indicated that EMT occurred in adenomyosis mice (Fig. 1d). Thus, our findings demonstrate that inactivation of the Hippo signaling pathway and EMT occurs in mice with adenomyosis.

### Verteporfin Affects Viability, Proliferation, and Apoptosis of Ishikawa Cells

**Fig. 2:**
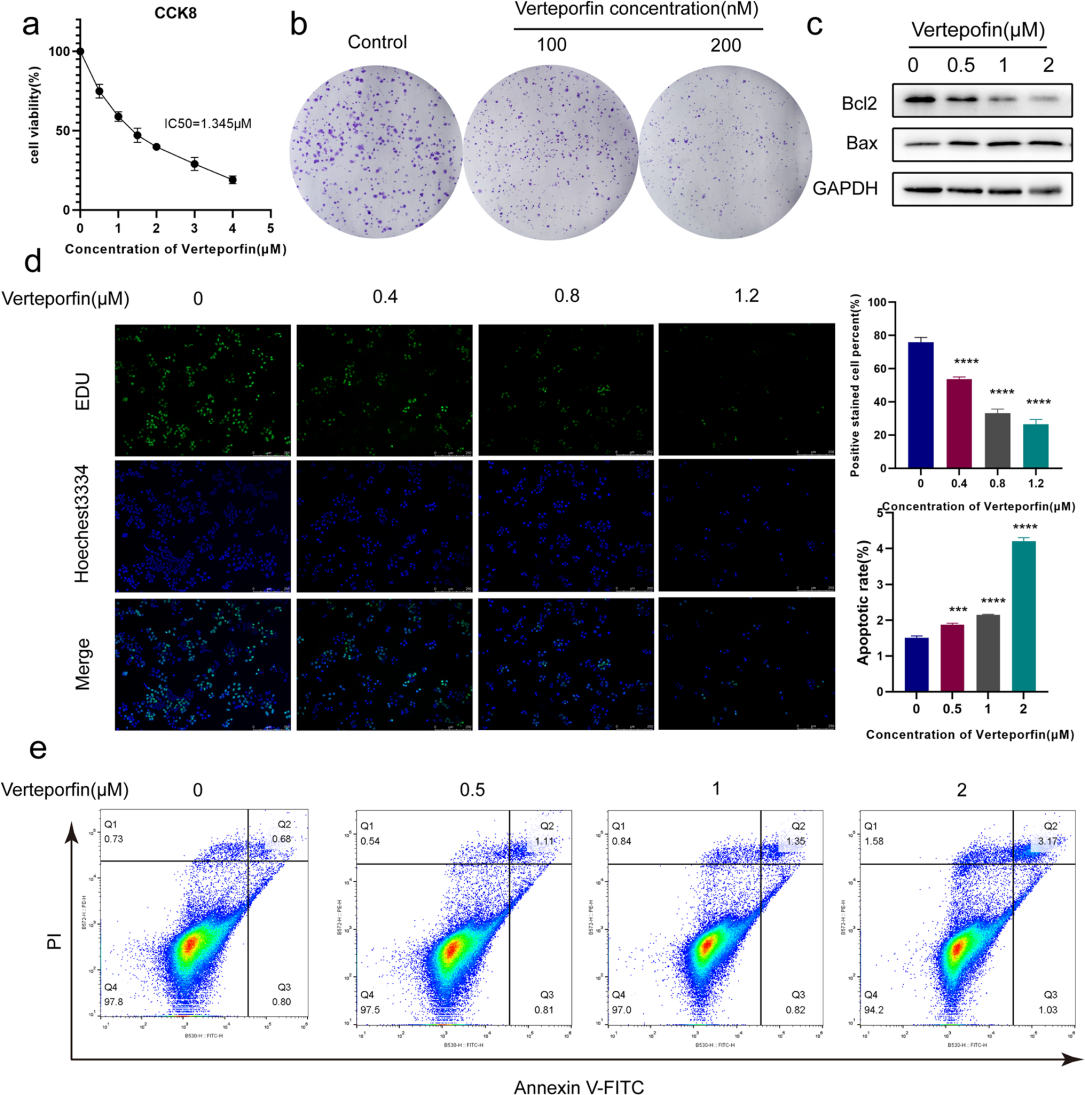
Verteporfin inhibits proliferation and promotes apoptosis of Ishikawa cells. **a**, **b**, and **d** CCK-8, colony formation, and EdU assay of the proliferation of verteporfin-treated Ishikawa cells. **c** Protein expression of bcl2 and bax in verteporfin-treated Ishikawa cells was determined by western blot. **e** Flow cytometry analysis of the apoptosis of verteporfin-treated Ishikawa cells. Data were presented as mean ± SD. ***P < 0.001; ****P < 0.0001

Ishikawa cells are a well-differentiated endometrial adenocarcinoma cell line and a good model for studying normal endometrial epithelial cells [8, 23–26]. We further used Ishikawa cells to study the regulatory relationship between the Hippo signaling pathway and EMT. Verteporfin is a specific YAP inhibitor that can inhibit tumor development by inhibiting the expression of YAP [27]. CCK-8, colony formation, EdU assay, and flow cytometry were used to analyze the viability, proliferation, and apoptosis of Ishikawa cells after verteporfin treatment. As shown in Fig. 2a, the cell viability gradually decreased with the increase of verteporfin concentration. The data also showed that the proliferation rate and the number of colony formation of cells were inhibited by verteporfin compared to the control group (Fig. 2b). In addition, we also found that the expression of anti-apoptotic marker B cell leukemia 2 (Bcl2) decreased and the expression of pro-apoptotic protein Bcl2-associated X protein (Bax) increased with the increase of verteporfin concentration (Fig. 2c). EdU staining also showed that inhibition of YAP reduced cell proliferation (Fig. 2d). Flow cytometry showed that the percentage of apoptotic cells was significantly increased in verteporfin-treated Ishikawa cells (Fig. 2e). These data suggest that activation of Hippo signaling pathway leads to reduced proliferation of endometrial epithelial cells and promotion of apoptosis.

### Verteporfin Inhibits the Migration Ability of Ishikawa Cells

**Fig. 3:**
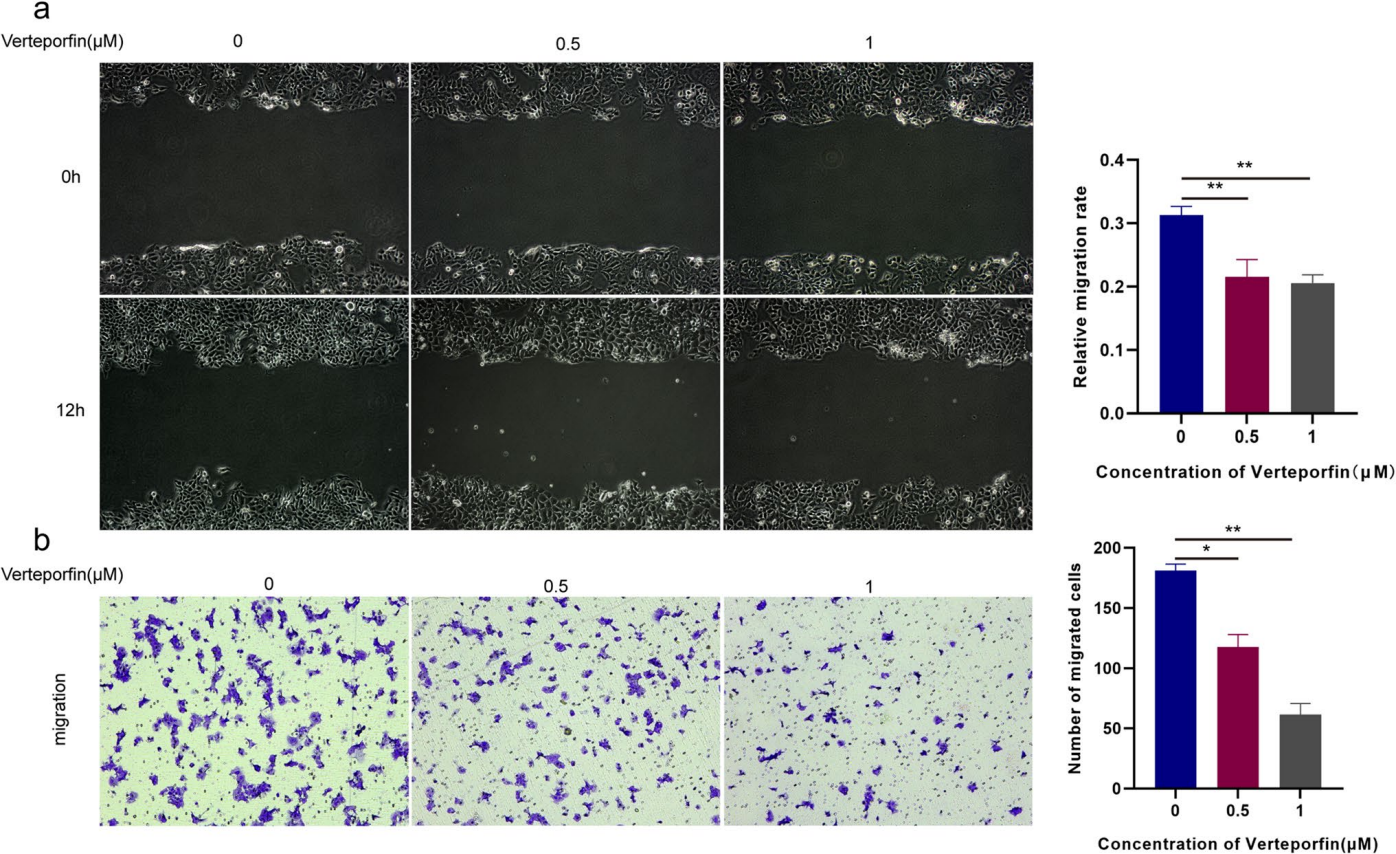
Verteporfin inhibits the migration ability of Ishikawa cells in vitro. **a** Wound healing assay of the migration ability of verteporfin-treated Ishikawa cells. **b** Transwell assay of the migration ability of verteporfin-treated Ishikawa cells. Data were presented as mean ± SD. *P < 0.05; **P < 0.01; ***P < 0.001

To examine the role of the Hippo signaling pathway in the development of adenomyosis, we sought to determine the effect of verteporfin on cell migration in vitro. As shown in Fig. 3, both wound healing assay and transwell assay results showed that the migration of Ishikawa cells was significantly reduced after verteporfin treatment, indicating that the Hippo signaling pathway plays an important role in mediating cell migration.

### Verteporfin Inhibits EMT Process of Ishikawa Cells

**Fig. 4:**
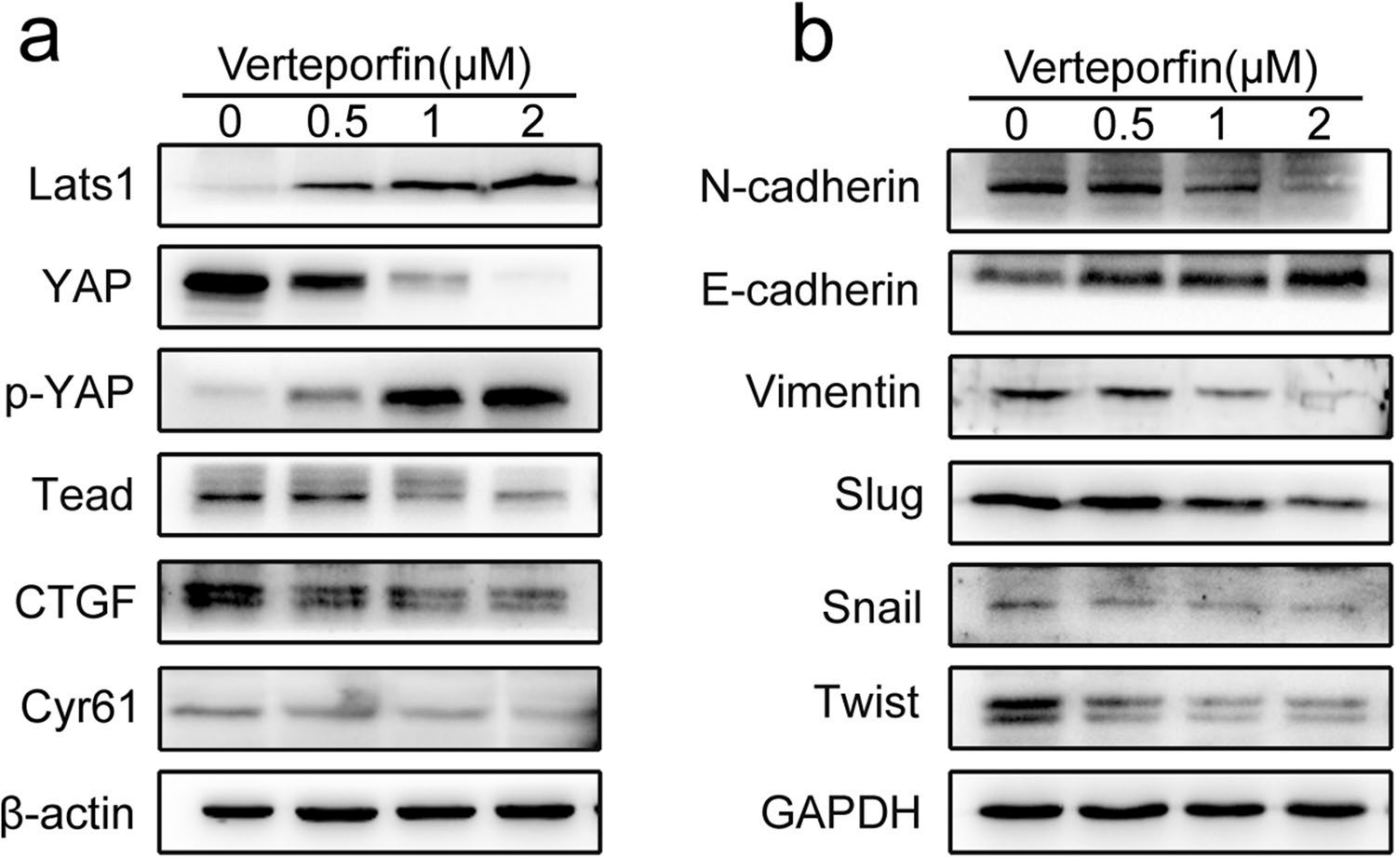
Verteporfin inhibits EMT process in Ishikawa cells. **a** Protein expression of Lats1, YAP, p-YAP, Tead, CTGF, and Cyr61 in the verteporfin-treated Ishikawa cells was determined by western blot. **b** Protein expression of E-cadherin, N-cadherin, Vimentin, Slug, Snail, and Twist in the verteporfin-treated Ishikawa cells was determined by western blot

To verify the regulation of Hippo signaling pathway on EMT in adenomyosis, we performed western blot to detect the expression of EMT marker proteins in verteporfin-treated Ishikawa cells. As shown in Fig. 4a, verteporfin activates the Hippo signaling pathway, which is manifested in the increased expression of Lats1 and p-YAP, and the decreased expression of YAP and Tead. At the same time, the expression of YAP target genes CTGF and Cyr61 decreased. Figure 4b shows that with the increase of verteporfin concentration, the EMT process was inhibited, which was reflected in the increased expression of E-cadherin and decreased expression of N-cadherin, Vimentin, Slug, Snail, and Twist. Collectively, these findings suggest that activation of Hippo signaling pathway suppresses the EMT process of Ishikawa cells.

### Inhibition of Hippo Signaling Pathway Partially Reverses EMT in Mice with Adenomyosis

**Fig. 5:**
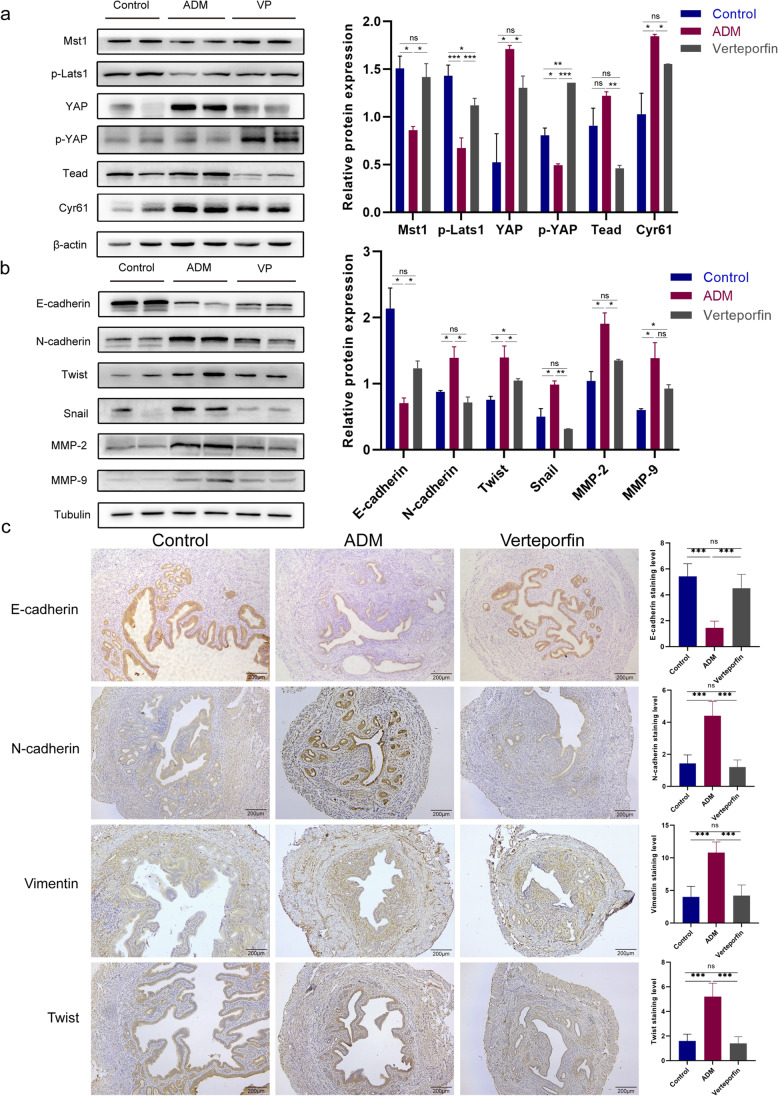
Verteporfin inhibits EMT process in mice with adenomyosis. **a** Protein expression of Mst1, p-Lats1, YAP, p-YAP, Tead, and Cyr61 was determined by western blot. **b** Protein expression of E-cadherin, N-cadherin, Twist, Snail, MMP-2, and MMP-9 was determined by western blot. **c** The expression levels of E-cadherin, N-cadherin, Vimentin, and Twist in uterine tissue were detected by IHC assay. Data were presented as mean ± SD. *n* = 5. *P < 0.05; **P < 0.01; ***P < 0.001; ns, no significance

To verify the regulatory relationship between the Hippo signaling pathway and EMT in adenomyosis, we injected the YAP inhibitor verteporfin into adenomyosis mice. As shown in Fig. 5a, the western blot results showed that the expression of Mst1, p-Lats1, and p-YAP was increased, and the expression of YAP, Tead, and Cyr61 was decreased in the verteporfin group compared with the adenomyosis group. These results demonstrate that verteporfin activates the Hippo signaling pathway in mice with adenomyosis.

After verifying that the Hippo signaling pathway was activated in the verteporfin group mice, we detected the expression of EMT-related proteins in the verteporfin group mice by western blot and IHC assay, and further studied the relationship between the Hippo signaling pathway and EMT in adenomyosis. As shown in Fig. 5b, compared with the adenomyosis group, the expression of E-cadherin was increased and the expression of N-cadherin, Twist, Snail, MMP-2, and MMP-9 was decreased in mice of verteporfin group. And the expression of EMT-related proteins detected by IHC further indicated that verteporfin inhibited the progression of EMT in adenomyosis mice (Fig. 5c). These data suggest that activation of the Hippo signaling pathway in adenomyosis can partially reverse the development of EMT, while reducing cell migration and invasion.

### Activation of Hippo Signaling Pathway Inhibits Cell Proliferation and Promotes Apoptosis in Mice with Adenomyosis

**Fig. 6:**
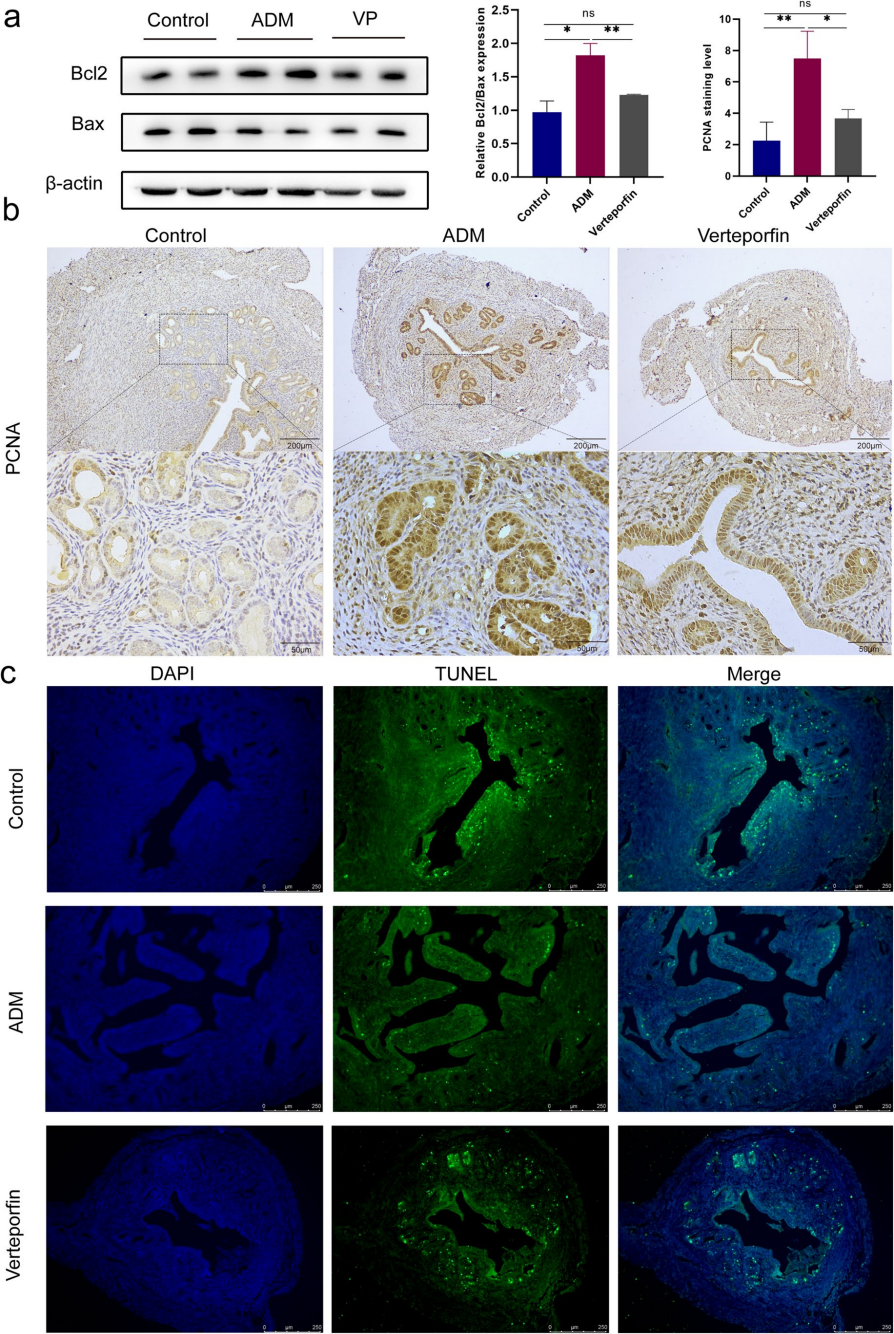
Verteporfin inhibits cell proliferation and promotes apoptosis in mice with adenomyosis. **a** Protein expression of Bcl2 and Bax was determined by western blot. **b** The expression level of PCNA in uterine tissue was detected by IHC assay. **c** The cell apoptosis in uterine tissue was detected by TUNEL assay. Data were presented as mean ± SD. *n* = 5. *P < 0.05; **P < 0.01; ns, no significance

The development of adenomyosis is accompanied by abnormal cell proliferation and apoptosis. As shown in Fig. 6a, western blot results showed that compared with the control mice, the expression of anti-apoptotic marker Bcl2 was increased and the expression of apoptosis protein Bax was decreased. Quantitative analysis showed that Bcl2/Bax was increased. In addition, we used the positive expression of proliferating cell nuclear antigen (PCNA) to identify cell proliferation in situ to detect cell proliferation. The level of PCNA positive staining in the uterine tissue of adenomyosis mice was significantly higher than that of control mice (Fig. 6b). These results suggest that abnormal proliferation occurs in mice with adenomyosis. At the same time, compared with the adenomyosis group, the expression of Bcl2 was decreased, the expression of Bax was increased, and Bcl2/Bax was decreased, while the expression of PCNA was decreased in the verteporfin group. These data demonstrate that activation of Hippo signaling pathway by verteporfin inhibits abnormal proliferation in adenomyosis mice relative to control mice.

TUNEL apoptosis test showed that TUNEL positive staining (green fluorescence) in adenomyosis mice was significantly lower than that in the control group, while the fluorescence intensity of verteporfin group was significantly higher than that of adenomyosis group, indicating that the number of apoptotic cells in adenomyosis mice was much lower than that in control mice and verteporfin treatment increased cell apoptosis in mouse uterine tissue (Fig. 6c). Therefore, these results suggest that activation of the Hippo signaling pathway by verteporfin in adenomyosis mice inhibits abnormal proliferation and promotes apoptosis of cells in adenomyosis mice.

## Discussion

Adenomyosis is an estrogen-dependent benign uterine disease with an incidence of about 20–30% worldwide [28], and the age of onset tends to be younger. Although the development of diagnostic techniques such as transvaginal ultrasonography (TVUS) and magnetic resonance imaging (MRI) has facilitated the clinical diagnosis of adenomyosis, the systematic basis for its pathogenesis is still lacking. Except for hysterectomy [29], there is currently no specific drug that can cure the disease, which brings great inconvenience to patients with fertility needs. There are many hypotheses about the pathogenesis of adenomyosis, one of which is generally accepted is the invagination theory. According to the invagination theory, endometrial tissue invades the myometrium mainly through the damaged endometrial-myometrial junction (JZ) [30]. Therefore, increased migratory and invasive abilities of endometrial cells may be a key factor in the development of adenomyosis. According to the available evidence, estrogen-induced epithelial-mesenchymal transition (EMT) provides endometrial cells with the ability to migrate and invade, which is critical for the pathogenesis of adenomyosis [12, 31]. In the current study, we found for the first time that the EMT occurring in adenomyosis may be regulated by the Hippo signaling pathway.

In recent years, YAP, as a key effector protein of the Hippo signaling pathway, has been identified to play an important role in the regulation of cell proliferation and apoptosis, tissue metabolism, organ growth and tumorigenesis, and development [32, 33]. Dysregulation of YAP has been found in a variety of diseases. Previous studies have shown that in various subtypes of breast cancer, the up-regulation of YAP expression can promote tumor cell proliferation, enhance the ability of tumor cells to metastasize, and make tumor cells resistant to chemotherapy [34]. Zhang et al. [35] showed that in glioma cells, the expression of mesenchymal markers N-cadherin and Twist was decreased after YAP knockdown and significantly increased after YAP overexpression. In addition, Zhang et al. [36] also reported that Furin can promote EMT through the Hippo signaling pathway in pancreatic cancer cells. In our study, we found that verteporfin inhibited the viability, proliferation, and migration and promoted apoptosis of Ishikawa cells in a concentration-dependent manner. Song et al. [14] found abnormal expression of YAP in endometriosis, and knockdown of YAP resulted in decreased cell proliferation and increased apoptosis in ESCs, which is consistent with our study. To confirm the relationship between the Hippo signaling pathway and EMT, the effect of verteporfin on EMT-related proteins in Ishikawa cells was investigated. Verteporfin treatment increased the expression level of E-cadherin and decreased the expression levels of N-cadherin, Vimentin, Twist, Snail, and Slug. These in vitro experiments demonstrated that activation of the Hippo signaling pathway could reduce the proliferation of endometrial epithelial cells, enhance cell apoptosis, and inhibit the occurrence of EMT.

EMT is known to promote the development of adenomyosis [37–39]. The result of EMT is often that cells acquire high migration and invasiveness. We found the occurrence of EMT in the uterine tissue of mice with adenomyosis in an in vivo study. Specifically, decreased expression of the epithelial marker E-cadherin and increased abundance of mesenchymal markers (i.e., N-cadherin, Vimentin, Twist, Snail) were recorded. At the same time, we also observed inactivation of the Hippo signaling pathway in adenomyosis mice, which is manifested in the high expression of the key protein YAP in adenomyosis. The study by Huang et al. [19] found that YAP is overexpressed in adenomyosis, which provides support for our findings. Subsequently, the inhibition of YAP by verteporfin injection in adenomyosis mice increased the expression level of E-cadherin and decreased the expression levels of N-cadherin, Twist, and Snail. Therefore, our findings suggest that EMT in adenomyosis is regulated by the Hippo signaling pathway.

Under the condition that EMT is regulated by Hippo signaling pathway in adenomyosis mice, changes in cell proliferation and apoptosis are the most intuitive phenotypes in the development of adenomyosis. The current study showed that the use of verteporfin to activate the Hippo signaling pathway increased apoptosis and inhibited proliferation of cells. This observation suggests that abnormal inactivation of the Hippo signaling pathway may promote cell proliferation and inhibit apoptosis, which in turn accelerates the development of adenomyosis.

In conclusion, both in vitro and in vivo experiments indicated that EMT and abnormal proliferation and apoptosis of cells in adenomyosis may be related to the inactivation of Hippo signaling pathway. Specifically, inactivation of the Hippo signaling pathway in adenomyosis has been proposed. Inhibition of YAP expression inhibits cell proliferation and promotes apoptosis in vitro and in vivo. EMT in adenomyosis may be related to the inactivation of Hippo signaling pathway, providing a reference for the underlying mechanism of adenomyosis.

## Data Availability

The data and material that support the findings of this study are available from the corresponding author upon reasonable request.

## Code Availability

Codes for data analysis are available upon request.
